# Pharmacogenomics on the Treatment Response in Patients with Psoriasis: An Updated Review

**DOI:** 10.3390/ijms24087329

**Published:** 2023-04-15

**Authors:** Ching-Ya Wang, Chuang-Wei Wang, Chun-Bing Chen, Wei-Ti Chen, Ya-Ching Chang, Rosaline Chung-Yee Hui, Wen-Hung Chung

**Affiliations:** 1 Department of Dermatology, Chang Gung Memorial Hospital, Linkou Branch, Taoyuan 333, Taiwan; 2 College of Medicine, Chang Gung University, Taoyuan 333, Taiwan; 3 Cancer Vaccine & Immune Cell Therapy Core Laboratory, Department of Medical Research, Chang Gung Memorial Hospital, Linkou Branch, Taoyuan 333, Taiwan; 4 Chang Gung Immunology Consortium, Chang Gung Memorial Hospital, Chang Gung University, Taoyuan 333, Taiwan; 5 Department of Dermatology, Xiamen Chang Gung Hospital, Xiamen 361028, China; 6 Immune-Oncology Center of Excellence, Chang Gung Memorial Hospital, Linkou Branch, Taoyuan 333, Taiwan; 7 Graduate Institute of Clinical Medical Sciences, College of Medicine, Chang Gung University, Taoyuan 333, Taiwan; 8 Whole-Genome Research Core Laboratory of Human Diseases, Chang Gung Memorial Hospital, Keelung 204, Taiwan; 9 Department of Dermatology, Chang Gung Memorial Hospital, Keelung Branch, Keelung 204, Taiwan; 10 Department of Dermatology, Beijing Tsinghua Chang Gung Hospital, School of Clinical Medicine, Tsinghua University, Beijing 100190, China; 11 Department of Dermatology, Ruijin Hospital, School of Medicine, Shanghai Jiao Tong University, Shanghai 200240, China; 12 Genomic Medicine Core Laboratory, Chang Gung Memorial Hospital, Linkou Branch, Taoyuan 333, Taiwan

**Keywords:** psoriasis, treatment response, adverse effect, pharmacogenetics, pharmacogenomics, polymorphisms, drug, whole genome sequencing

## Abstract

The efficacy and the safety of psoriasis medications have been proved in trials, but unideal responses and side effects are noted in clinical practice. Genetic predisposition is known to contribute to the pathogenesis of psoriasis. Hence, pharmacogenomics gives the hint of predictive treatment response individually. This review highlights the current pharmacogenetic and pharmacogenomic studies of medical therapy in psoriasis. HLA-Cw*06 status remains the most promising predictive treatment response in certain drugs. Numerous genetic variants (such as ABC transporter, DNMT3b, MTHFR, ANKLE1, IL-12B, IL-23R, MALT1, CDKAL1, IL17RA, IL1B, LY96, TLR2, etc.) are also found to be associated with treatment response for methotrexate, cyclosporin, acitretin, anti-TNF, anti-IL-12/23, anti-IL-17, anti-PDE4 agents, and topical therapy. Due to the high throughput sequencing technologies and the dramatic increase in sequencing cost, pharmacogenomic tests prior to treatment by whole exome sequencing or whole genome sequencing may be applied in clinical in the future. Further investigations are necessary to manifest potential genetic markers for psoriasis treatments.

## 1. Introduction

Psoriasis is a chronic, immune-mediated, inflammatory skin disease concomitant with other systemic complications. Environmental, behavioral, and genetic factors play a role in the etiology of the disease. Especially, genetic predisposition is thought to be a key contributor to psoriasis through involvement in immune pathophysiology [1], and about 40% of patients diagnosed with psoriasis or psoriatic arthritis have a related family history [2]. To date, almost 100 psoriasis susceptibility loci have been identified through selective candidate genes or genome-wide association studies (GWAS) [3]. The pharmacogenetic issue of psoriasis struck a chord after the immunogenetics of psoriasis were outlined gradually, and the need for personalized medicine increased when more and more anti-psoriatic drugs were available and showed variable efficacy among different drugs and individuals. This study aimed to overview the current findings of possible genetically predictive markers for treatment outcomes of psoriasis under the use of systemic and topical medicine.

## 2. Pathophysiology and Immunogenetics

**Figure 1:**
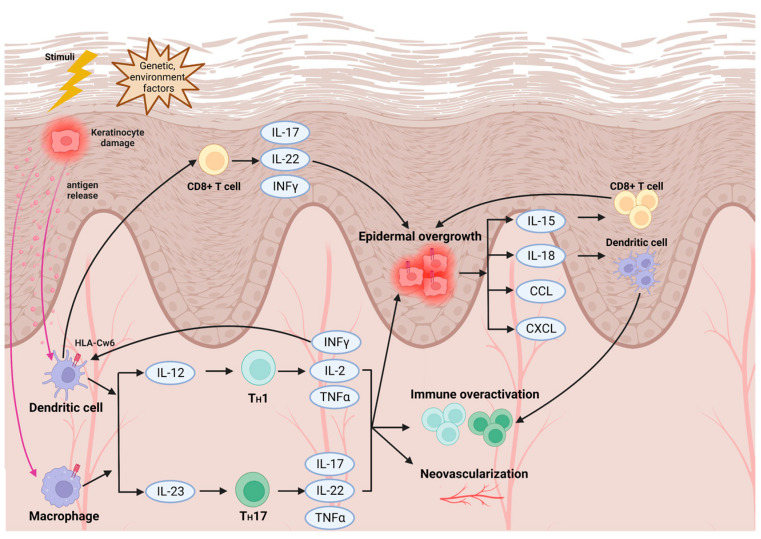
Immunopathogenesis of psoriasis.

Regards to pathogenesis and immunogenetics of psoriasis (Figure 1), the disease results from an aberrant innate or adaptive immune response associated with T lymphocytes that leads to inflammation, angiogenesis, and epidermal hyperplasia [4].

Genetic or environmental factors can trigger immune-mediated damage for keratinocytes in psoriasis patients. The key pathomechanism of psoriasis is that dendritic cells or macrophages can secrete IL-23 and then stimulate CD4^+^Th17 polarization, resulting in the secretion of cytokines, such as IL-17, IL22, TNF-α, etc. Moreover, IL-12 can activate the differentiation of CD4^+^Th1 cells, which induces INF-γ, IL-2, and TNF-α synthesis; CD8^+^ T cells are also known to be activated and can release pro-inflammatory cytokines, including TNF-α and INF-γ. The abundant cytokines lead to epidermal overgrowth, immune over-activation, and neovascularization. Consequently, the positive feedback loop of immune reaction leads to the development and maintenance of psoriatic lesions.

The initiation of psoriasis lesion is when antigenic or auto-antigenic stimuli induced by damaged or stressed skin activate antigen-presenting cells (APCs), including dendritic cells (DCs) and macrophages. The process results in producing pro-inflammatory cytokines such as interferon (IFN)-α, tumor necrosis factor (TNF)-α, interleukin (IL)-12, IL-20, and IL-23, and initiates the early phase of cutaneous inflammation in psoriasis [5].

The pro-inflammatory cytokines released from activated APCs promote T cell-mediated immunity through nuclear factor kappa-light-chain-enhancer of activated B cells (NF-κB) pathway and Janus kinase (JAK)-signal transducer and activator of transcription (STAT) pathway. In addition, engagement of the T cell receptor (TCR) with major histocompatibility complex (MHC)-presenting antigen of APCs activates the calcium–calcineurin–nuclear factor of activated T cells (NFAT) pathway. Thus, these signals result in the migration, differentiation, and activation of naïve effector T cells. In particular, IL-23 stimulates CD4^+^T helper 17 (Th17) polarization, which releases IL-17A/F, IL-22, and TNF-α. On the other hand, IL-12 activates the differentiation of the Th1 subset of CD4+ cells, which induces INF-γ, IL-2, and TNF-α synthesis [6].

The inflammatory cytokines secreted from T cells, especially IL-17A, attract many more immune cells, such as neutrophils, enhance angiogenesis, facilitate hyperproliferation of keratinocytes, and promote the further release of cytokines. Additionally, keratinocytes activated by IL-17, IL-22, and IL-20 through JAK-STAT, NF-κB, and calcium–calcineurin–NFAT pathways release C-C motif ligand 20 (CCL20), antimicrobial peptides (AMP), and cytokines; hence, they contribute to the pro-inflammatory environment and amplify the inflammatory response [7].

In brief, the over-activated innate immunity induces exaggerative T cell-mediated autoimmune activation, epidermal overgrowth, and neovascularization. Consequently, a positive feedback loop leads to the development and maintenance of psoriatic lesions. The psoriasis susceptibility genes were found to involve in the entire immunopathogenesis from antigen presentation, cytokines and receptors, signal transductions, and transcription factors to regulators of immune responses [1,8]; at the same time, whether these susceptibility genes are potential predictors of treatment response has been investigated. In the following context, we discuss the response-related genes in psoriasis treatment (Table 1, Table 2, Table 3, Table 4, Table 5, Table 6 and Table 7, Supplementary Table S1) and present levels of evidence of the pharmacogenomic association by the PharmGKB annotation scoring system. According to PharmGKB, six levels from 1A to 4 represent high, moderate, and low to unsupported evidence, respectively.

## 3. Treatment

### 3.1. Methotrexate

Methotrexate (MTX) is an antagonist of the enzymes dihydrofolate reductase (DHFR) and thymidylate synthase (TYMS). It is commonly used as a first-line systemic immunosuppressive therapy for moderate to severe psoriasis. However, significant variations in its efficacy and toxicity exist among individuals. Therefore, several studies have identified potential pharmacogenetic factors that can be used to predict the clinical response of MTX (Table 1).

#### 3.1.1. ABCC1, ABCC2, ABCG2

The genes encoding the efflux transporters of MTX are *ATP-binding cassette (ABC) subfamily C member 1 (ABCC1)*, *ABCC member 2 (ABCC2)*, and *ABC subfamily G member 2 (ABCG2)*. Overexpression of these genes can lead to multidrug resistance by extruding drugs out of the cell through various mechanisms [9,10]. In regard to psoriasis, a cohort study of 374 British patients found significant positive associations between methotrexate responder, two of *ABCG2* (rs17731538, rs13120400), and three SNPs of *ABCC1* (rs35592, rs28364006, rs2238476) with rs35592 being the most significant (PASI75 at 3 months, *p* = 0.008). One cohort study from Slovenia demonstrated that polymorphism of *ABCC2* (rs717620) presented an insufficient response to MTX treatment (75% reduction from baseline PASI score (PASI75) at 6 months, *p* = 0.039) [11]. About toxicity, a British cohort study has noted that the major allele of six SNPs in *ABCC1* (rs11075291, rs1967120, rs3784862, rs246240, rs3784864, and rs2238476) was significantly associated with the onset of adverse events, with rs246240 showing the strongest association (*p* = 0.0006) [12].

#### 3.1.2. ADORA2A

*Adenosine receptors A2a (ADORA2a)* is responsible for mediating the metabolic product of methotrexate. One SNP, rs5760410 of *ADORA2A*, was weakly associated with the onset of toxicity (*p* = 0.03) [12].

#### 3.1.3. ATIC

MTX inhibits *5-aminoimidazole-4-carboxamide ribonucleotide formyltransferase (ATIC)*, which leads to the accumulation of adenosine, a potent anti-inflammatory agent [13].

Campalani et al. analyzed 188 patients in the United Kingdom (UK) with psoriasis under methotrexate therapy and revealed that allele frequency of *ATIC* (rs2372536) was significantly increased in patients who discontinued methotrexate owing to intolerable side effects (*p* = 0.038) [14]. Another British cohort study found that two SNPs in *ATIC* (rs2372536 and rs4672768) were associated with the onset of MTX toxicity (*p* = 0.01). However, these associations did not remain significant after adjusting for folic acid supplementation [15].

#### 3.1.4. BHMT

*Betaine-homocysteine S-methyltransferase (BHMT)* is a zinc-containing metalloenzyme responsible for folate-independent remethylation of homocysteine using betaine as the methyl donor [16]. A genotype analysis identified that the *BHMT* genotype was significantly associated with MTX hepatotoxicity (*p* = 0.022) [11].

#### 3.1.5. DNMT3b

*DNA methyltransferase 3β (DNMT3b) is* a methyltransferase that is involved in de-novo DNA methylation, and its polymorphism is supposed to be associated with increased promoter activity [17]. At least one copy of the variant *DNMT3b* rs242913 allele has been found to be associated with an insufficient response to MTX when compared to the wild-type (*p* = 0.005) [11].

#### 3.1.6. FOXP3

*Forkhead box P3 (FOXP3)* appears to function as a master regulator of the regulatory pathway in the development and function of regulatory T cells (Tregs) [18]. A study on a population of 189 southern Indian patients who had used methotrexate for 12 weeks found a significant difference in genotype frequencies of *FOXP3* (rs3761548) between responders and non-responders (PASI75 at 3 months, *p* = 0.003) [19].

#### 3.1.7. GNMT

*Glycine N-methyltransferase (GNMT)* is a methyltransferase that converts S-adenosylmethionine to S-adenosylhomocysteine and is also a folate-binding protein. The rs10948059 polymorphism is associated with increased expression of the *GNMT* gene and reduces cell sensitivity to MTX [20]. The patients with at least one variant GNMT allele were more likely to be non-responders to MTX treatment than the reference allele (PASI75 at 6 months, *p* = 0.0004) [11].

#### 3.1.8. HLA-Cw6

The *human leukocyte antigen (HLA)*, known as the human MHC system, regulates the immune system by encoding cell-surface proteins. *HLA-Cw6* is a psoriasis susceptibility allele that has been strongly linked to the disease. It was reported that carriers of HLA-Cw6 from southern India had a higher response rate to methotrexate (PASI75 at 3 months, *p* = 0.003) [19]. A Scotland cohort study with 70 HLA-tested patients demonstrated that more proportion of *HLA-Cw6* positive patients was carried on beyond 12 months, as compared to the HLA-Cw6 negative group (*p* = 0.05) [21].

#### 3.1.9. MTHFR

The Methylenetetrahydrofolate reductase (MTHFR) enzyme is responsible for catalyzing the formation of 5-methyl-tetrahydrofolic acid, which acts as a methyl donor for the synthesis of methionine from homocysteine. This enzyme is indirectly inhibited by MTX. [22] According to Zhu et al., the PASI 90 response rates to MTX were significantly higher in Han Chinese patients who had the MTHFR rs1801133 TT genotype as compared to those who had the CT and CC genotype (PASI90 at 3 months, *p* = 0.006). Furthermore, patients with the *MTHFR* rs1801131 CT genotype had lower PASI 75 response rates to MTX in Han Chinese population (PASI75 at 3 months, *p* = 0.014). They also had a lower risk of ALT elevation (*p* = 0.04) [23]. However, three studies have demonstrated that no significant association was detected between clinical outcomes in individuals with psoriasis treated with methotrexate and SNPs in the *MTHFR* gene [11,14,15].

#### 3.1.10. SLC19A1

The *Solute carrier family 19*, *member 1 (SLC19A1)* gene encodes the reduced folate carrier (RFC) protein, which actively transports MTX into cells. Multiple point mutations have been identified in *SLC19A1* to be associated with impaired MTX transport and resistance to MTX [24]. *SLC19A1* (rs1051266) was associated with MTX-induced toxicity instead of efficacy in patients with psoriasis [12,14].

#### 3.1.11. SLCO1B1

The encoded protein of *solute carrier organic anion transporter family member 1B1 (SLO1B1)* is a transmembrane receptor that transports drug compounds into cells. Genetic variations in *SLCO1B1* have been linked to delayed MTX clearance and increased toxicity [25,26]. The haplotype variants have been classified into two groups based on their reported transporter activity: the high-activity group and the low-activity group. Patients with low-activity haplotypes of SLCO1B1 (SLCO1B1*5 and SLCO1B1*15) were less likely to be MTX non-responders as compared to patients with high-activity haplotypes (SLCO1B1*1a and SLCO1B1*1b) (PASI75 at 6 months, *p* = 0.027) [11].

#### 3.1.12. TNIP1

*TNFAIP3 interacting protein 1 (TNIP1)*, as one of the psoriasis susceptibility genes, is related to the immune response IL-23 signaling pathway. A Chinese study mentioned that in 221 patients with psoriasis, the TT genotype of *TNIP1* rs10036748 showed a better response to MTX (PASI75 at 3 months, *p* = 0.043) [27].

#### 3.1.13. TYMS

Thymidylate synthase (TS), encoded by the *thymidylate synthase gene (TYMS)*, is a critical protein for pyrimidine synthesis and responsible for DNA synthesis and repair, which could be inhibited by MTX [28]. The association of polymorphisms of *TYMS*, TS levels, and MTX response was found in several diseases [29,30]. For example, polymorphism rs34743033 is a 28-base pair (bp) with double or triple tandem repeat (2R or 3R) located on the 5′ untranslated region (UTR) [31]. A study performed in European adults with psoriasis found that the rs34743033 3R allele was more frequent in patients with poor therapeutic response to methotrexate, but the loss of significance was noted after the exclusion of palmoplantar pustulosis patients. In addition, this allele was significantly associated with an increased incidence of MTX-induced toxicity in patients who did not receive folic acid (*p* = 0.0025). Another TS polymorphism, 3′-UTR 6bp del of rs11280056, was significantly more frequent in patients with an adverse event irrespective of folic acid supplementation (*p* = 0.025) [14].

In short, positive genotypic associations were detected with methotrexate responders in ten genes (*ABCC1*, *ABCC2*, *ABCG2*, *DNMT3b*, *FOXP3*, *GNMT*, *HLA-Cw*, *MTHFR*, *SLCO1B1*, *TNIP1*) while the development of methotrexate-related toxicity in five genes (*ABCC1*, *ATIC*, *ADORA2A*, *BHMT*, *MTHFR*, *SLC19A1*, *TYMS*). Nonetheless, three British studies seemed to believe that toxicity has overlapped populations; hence, several replicated results may also be owing to similar databases [14,15,22].

### 3.2. Acitretin

Acitretin is an oral vitamin A derivative that is used to treat psoriasis by inhibiting epidermal proliferation, inflammatory processes, and angiogenesis. Table 2 lists the genetic polymorphisms that have been associated with the response of acitretin in patients with psoriasis.

#### 3.2.1. ApoE

Apolipoprotein E (ApoE) is a glycoprotein component of chylomicrons and VLDL. It has a crucial role in regulating lipid profiles and metabolism [32]. The lipid and lipoprotein abnormalities as a consequence of *ApoE* gene polymorphism are close to the side effects during acitretin therapy. In addition, *ApoE* levels have been linked with clinical improvement in psoriasis, indicating a potential role of the gene in acitretin treatment for psoriasis [33]. However, according to Campalani, E, et al., while ApoE gene polymorphisms are associated with psoriasis, they do not determine the response of the disease to acitretin [34].

#### 3.2.2. ANKLE1

Ankyrin repeat and LEM domain containing 1 (ANKLE1) enables endonuclease activity and plays a role in positively regulating the response to DNA damage stimulus and protein export from the nucleus. *ANKLE1* rs11086065 AG/GG was associated with an ineffective response compared to the GG genotype in 166 Chinese patients (PASI75 at 3 months, *p* = 0.003) [35].

#### 3.2.3. ARHGEF3

Rho guanine nucleotide exchange factor 3 (ARHGEF3) activates Rho GTPase, which involve in bone cell biology. *ARHGEF3* rs3821414 CT was associated with a more effective response compared to the TT genotype (PASI75 at 3 months, *p* = 0.01) [35].

#### 3.2.4. CRB2

*Crumbs cell polarity complex component 2 (CRB2)* encodes proteins that are components of the Crumbs cell polarity complex, which plays a crucial role in apical-basal epithelial polarity and cellular adhesion. *CRB2* rs1105223 TT/CT was also associated with acitretin efficacy compared to the CC genotype (PASI75 at 3 months, *p* = 0.048) [35,36].

#### 3.2.5. HLA-DQA1*02:01

HLA-DQA1*0201 alleles may act as psoriasis susceptibility genes or may be closely linked to the susceptibility genes in Han Chinese [36]. Among 100 Chinese individuals, those who were positive for the *DQA10201* allele demonstrated a more favorable response to acitretin compared to those who were negative for the same allele. (PASI75 at 2 months, *p* = 0.001) [37].

#### 3.2.6. HLA-DQB1*02:02

*HLA-DQB1* alleles have been mentioned to involve in genetic predisposition to psoriasis vulgaris in the Slovak population [38]. In 100 Chinese patients, the DQB1*0202-positive patients showed a better response to acitretin than the DQB1*0202-negative patients (PASI75 at 2 months, *p* = 0.005) [37].

#### 3.2.7. HLA-G

*HLA-G* is a nonclassical class I MHC molecule that plays a role in suppressing the immune system by inhibiting natural killer cells and T cells [39]. Among patients treated with acitretin, Borghi, Alessandro, et al. observed a significantly increased frequency of the 14 bp sequence deletion in the exon 8 of the *HLA-G* allele, functioning as a modification of mRNA stability, in responder patients, in comparison to the non-responders (PASI75 at 4 months, *p* = 0.008) [40].

#### 3.2.8. IL-12B

Patients with the *IL-12B* rs3212227 genotype of TG were more responsive to acitretin in the treatment of psoriasis in 43 Chinese patients (PASI50, *p* = 0.035) [41].

#### 3.2.9. IL-23R

Acitretin was found to improve the secondary non-response to TNFα monoclonal antibody in patients who were homozygous for the AA genotype at the SNP rs112009032 in the *IL-23R* gene (PASI75, *p* = 0.02) [41].

#### 3.2.10. SFRP4

Secreted frizzled-related protein 4 (SFRP4) is a negative regulator of the Wnt signaling pathway, and the downregulation of SFRP4 is a possible mechanism contributing to the hyperplasia of the epidermis of psoriasis [42]. The GG/GT variation of *SFRP4* rs1802073 has been found to be associated with a more effective response to acitretin compared to the TT genotype (PASI75 at 3 months, *p* = 0.007) [35,36].

#### 3.2.11. VEGF

*Vascular endothelial growth factor (VEGF)* promotes angiogenesis in the pathophysiology of psoriasis, and the variant of the *VEGF* gene is supposed to affect the ability of acitretin to downregulate *VEGF* production [43]. The TT genotype of the *VEGF* rs833061 was associated with non-response to oral acitretin, whereas the TC genotype was associated with a significant response to acitretin (PASI75 at 3 months, *p* = 0.01) [44]. However, the result of *VEGF* polymorphism was not replicated in the population of southern China [45].

### 3.3. Cyclosporin

Cyclosporine, a calcineurin inhibitor, is commonly used to treat moderate to severe psoriasis. However, clinical studies investigating the pharmacogenetics of cyclosporine in psoriasis patients are currently lacking (Table 3).

#### 3.3.1. ABCB1

One Greek study enrolled 84 patients revealed that *ATP-binding cassette subfamily B member 1 (ABCB1)* rs1045642 had statistically significant association with a negative response of cyclosporin (PASI < 50 at 3 months, *p* = 0.0075) [46]. In 168 Russian patients with psoriasis receiving cyclosporine therapy, a strongly negative association was observed for the TT/CT genotype of *ABCB1* rs1045642 (PASI75 at 3 months, *p* < 0.001), the TT/CT genotype of *ABCB1* rs1128503 (PASI75 at 3 months, *p* = 0.027), and the TT/GT genotype of *ABCB1* rs2032582 (PASI75 at 3 months, *p* = 0.048), respectively. Additionally, the TGC haplotype was significantly linked to a negative response (PASI75 at 3 months, *p* < 0.001) [47].

#### 3.3.2. CALM1

Calmodulin (CALM1) is known as a calcium-dependent protein and is related to cell proliferation and epidermal hyperplasia in psoriasis [48]. In 200 Greek patients, the allele T of *CALM1* rs12885713 displayed a significantly better response to cyclosporin (PASI75 at 3 months, *p* = 0.011) [49].

#### 3.3.3. MALT1

*MALT1* encodes MALT1 paracaspase, a potent activator of the transcription factors NF-κB and AP-1, and hence has a role in psoriasis [50]. MALT1 rs287411 allele G was associated with the effective response compared to allele A (PASI75 at 3 months, *p* < 0.001) [49].

### 3.4. Tumor Necrosis Factor Antagonist

There are four FDA-approved TNF antagonists for plaque psoriasis, including etanercept, adalimumab, infliximab, and certolizumab pegol. According to our review of the literature, pharmacogenetic research has been mainly focused on the first three drugs. Etanercept is a recombinant fusion protein comprising two extracellular parts of the human tumor necrosis factor receptor 2 (TNFR2) coupled to a human immunoglobulin 1 (IgG1) Fc. Adalimumab is a fully human monoclonal antibody with human TNF binding Fab and human IgG1 Fc backbone, whereas infliximab is a chimeric IgG1 monoclonal antibody composed of a human constant and a murine variable region binding to TNFα [51]. Despite their unique pharmacological profile from each other, TNF antagonists act on the same pathologic mechanism to achieve therapeutic outcomes. Therefore, some pharmacogenetic researchers regarded all TNF antagonists as one category to analyze potential predictive genetic markers under a large-scale population, while some discussed each TNF antagonist separately (Table 4).

#### 3.4.1. Nonspecific TNF Antagonist

##### Better Response of Efficacy

In 144 Spanish patients, carriers of the CT/CC allele in *MAP3K1* rs96844 and the CT/TT allele in *HLA-C* rs12191877 achieved a better PASI75 response at 3 months. The study also found significantly better results for carriers of *MAP3K1* polymorphism and CT/TT in *CDKAL1* rs6908425 at 6 months [52]. Another study enrolled 70 patients in Spain implicated that patients harboring high-affinity alleles, *FCGR2A-H131R* (rs1801274) and *FCGR 3A-V158F*(rs396991), contribute to better mean BSA improvement but not PASI improvement at 6–8 weeks after anti-TNF treatment of psoriasis [53]. The result between *FCGR 3A-V158F*(rs396991) and response to anti-TNFα therapy (PASI75 at 6 months, *p* = 0.005), especially etanercept (PASI75 at 6 months, *p* = 0.01), was replicated in 100 Caucasian patients from Greece, while *FCGR2A-H131R* (rs1801274) was found to be no association [54]. A study conducted in 199 Greek patients found an association between carriers of CT/CC in *HLA-C* rs10484554 and a good response to anti-TNF agents (PASI 75 at 6 months, *p* = 0.0032), especially adalimumab (*p* = 0.0007) [55].

In 238 Caucasian adults in Spain, the rs4819554 promoter SNP allele A of the *IL17RA* gene was significantly more prevalent among responders at week 12 [56]. Moreover, several genetic variants exert favorable effects at 6 months of treatment in 109 patients with psoriasis from Spain, including GG genotype of *IL23R* rs11209026 (PASI90 *p* = 0.006), GG genotype of TNF-a-238 rs361525 (PASI75, *p* = 0.049), CT/TT genotypes of TNF-a-857 rs1799724 (PASI75, *p* = 0.006, ΔPASI, *p* = 0.004; BSA, *p* = 0.009), and TT genotype of TNF-a-1031 rs1799964 (PASI75, *p* = 0.038; ΔPASI, *p* = 0.041; at 3 months, PASI75, *p* = 0.047) [57].

##### Poor Response of Efficacy

In 144 Spanish patients, four SNPs were associated with the inability to achieve PASI75 at three months, including AG/GG allele in *PGLYRP4-24* rs2916205, CC allele in *ZNF816A* rs9304742, AA allele in *CTNNA2* rs11126740, and AG/GG allele in *IL12B* rs2546890. Additionally, the results for polymorphisms in the *IL12B* gene were replicated at six months and one year. The study also obtained significant results for the *FCGR2A* and *HTR2A* polymorphism at 6 months [52]. Notably, the result of the *FCGR2A* polymorphism showed variability between studies [52,53,54]. In 376 Danish patients, five SNPs, which are *IL1B* (rs1143623, rs1143627), *LY96* (rs11465996), and *TLR2* (rs11938228, rs4696480), were all associated with nonresponse to treatment [58]. One study found a higher frequency of G-carriers of the *TNFRSF1B* rs1061622 among non-responders (PASI < 50) compared to cases achieving PASI75 to TNF blockers in 90 Caucasians from Spain [59].

##### Toxicity

Among the 161 Caucasian patients, the polymorphism rs10782001 in *FBXL19* and rs11209026 in *IL23R* may contribute to an increased risk of the secondary development of psoriasiform reactions owing to TNF blocking. In addition, in 70 Spanish patients, the copy number variation (CNV) harboring three genes (ARNT2, LOC101929586, and MIR5572) was related to the occurrence of paradoxical psoriasiform reactions at 3 and 6 months (*p* = 0.006) [60]. In contrast, the presence of rs3087243 in *CTLA4*, rs651630 in *SLC12A8*, or rs1800453 in *TAP1* was related to protection against psoriasiform lesions [61]. Interestingly, the *IL23R* rs11209026 polymorphism was reported as having a protective role reported in classical psoriasis.

#### 3.4.2. Etanercept (ETA/ETN)

##### CD84

*Cluster of Differentiation 84 (CD84)* gene encodes a membrane glycoprotein, which enhances IFN-γ secretion in activated T cells [62]. In 161 patients from the Netherlands, the GA genotype in *CD84* (rs6427528) had a more sensitive response to etanercept than the referential GG genotype (ΔPASI at 3 months, *p* = 0.025) [63].

##### FCGR3A

This gene encodes a receptor for the Fc portion of immunoglobulin G, where the TNF antagonist binds specifically. In 100 psoriasis patients in Greece, the study showed an association with *FCGR3A-V158F* (rs396991) and better response to etanercept (PASI75 at 6 months, *p* = 0.01) [54].

##### TNFAIP3

*TNFα induced protein 3 (TNFAIP3)* plays a protective role against the harmful effects of inflammation and is involved in immune regulation [64]. Rs610604 in *TNFAIP3* showed associations with good responses to etanercept (PASI75 at 6 months, *p* = 0.007) [55].

##### TNF, TNFRSF1B

TNFα transmits signals through *TNF receptor superfamily member 1B (TNFRSF1B)*, which exhibits predominantly on Tregs and is responsible for initiating immune modulation [65]. Carriage of *TNF-857C* (rs1799724) or *TNFRSF1B-676T* (rs1061622) alleles was associated with a positive response to drug treatment in patients treated with etanercept (PASI75 at 6 months, *p* = 0.002 and *p* = 0.001, respectively) [66].

#### 3.4.3. Adalimumab (ADA) & Infliximab (IFX/INF)

##### *CPM* 

CPM (Carboxypeptidase M) is involved in the maturation of macrophages in psoriasis pathogenesis [67]. The CNV of the *CPM* gene was significantly associated with adalimumab response among 70 Spanish patients (PASI75 at 3 and 6 months, *p* < 0.05) [60].

##### HLA

The rs9260313 in the *HLA-A* gene was found to be associated with more favorable responses to adalimumab (PASI75 at 6 months, *p* = 0.05) [55]. Among 169 Spanish patients, *HLA-Cw06* positivity had a better response to adalimumab. (PASI75 at 6 months, *p* = 0.018) [68].

##### IL17F

*IL-17F*, activated by IL23/Th17, is recognized as having a critical role in the pathogenesis of psoriasis. In a cohort study in Spain, carriers of TC genotype in *IL-17F* rs763780 were associated with a lack of response to adalimumab (*n* = 67, PASI75 at weeks 24–28, *p* = 0.0044) while interestingly, with better response to infliximab (*n* = 37, PASI at weeks 12–16, *p* = 0.023; PASI at weeks 24–28, *p* = 0.02).

##### NFKBIZ

The *nuclear factor of kappa light polypeptide gene enhancer in B cells inhibitor*, *zeta (NFKBIZ)* gene encodes an atypical inhibitor of nuclear factor κB (IκB) protein, involved in inflammatory signaling of psoriasis [69]. Among 169 Spanish patients, the deletion of *NFKBIZ* rs3217713 had a better response to adalimumab (PASI75 at 6 months, *p* = 0.015) [68].

##### TNF, TNFRSF1B

None of the genotyped SNPs of *TNF*, *TNFRSF1A*, *and TNFRSF1B* genes were associated with responsiveness to treatment with infliximab or adalimumab [66].

##### TRAF3IP2

TNF receptor-associated factor 3 interacting protein 2 (TRAF3IP2) involves in IL-17 signaling and interacts with members of the Rel/NF-κB transcription factor family [70]. The rs13190932 in the *TRAF3IP2* gene showed associations with a favorable response to infliximab (PASI75 at 6 months, *p* = 0.041) [55].

### 3.5. IL-12/IL-23 Antagonist

Ustekinumab, as an IL12/IL23 antagonist, targets the p40 subunit that is shared by IL-12 and IL-23, whereas guselkumab, tildrakizumab, and risankizumab target the p19 subunit of IL-23. These four drugs are efficacious in treating moderate to severe plaque psoriasis [71]. While ustekinumab is the earliest commercially available drug among IL23 antagonists, relatively abundant studies of the association between the response and gene status have been conducted. In contrast, there is limited research on the genetic predictors of clinical response to guselkumab, tildrakizumab, and risankizumab (Table 5).

#### 3.5.1. Ustekinumab (UTK)

##### Better Response of Efficacy

In a Spanish study enrolled 69 patients, good responders at 4 months were associated with CC genotype in *ADAM33* rs2787094 (*p* = 0.015), CG/CC genotype in *HTR2A* rs6311 (*p* = 0.037), GT/TT genotype in *IL-13* rs848 (*p* = 0.037), CC genotype in *NFKBIA* rs2145623 (*p* = 0.024), and CT/CC genotype in *TNFR1* rs191190 [72]. Rs151823 and rs26653 in the *ERAP1* gene showed associations with a favorable response to anti-IL-12/23 therapy among 22 patients from the UK. [55] Several studies exhibited that the presence of the HLA-Cw*06 or Cw*06:02 allele may serve as a predictor of faster response and better response to ustekinumab in Italian, Dutch, Belgian, American, and Chinese patients [72,73,74,75,76,77]. A recent meta-analysis study confirmed that HLA-C*06:02-positive patients had higher response rates (PASI76 at 6 months, *p* < *0*.001) [78]. In addition, the presence of the GG genotype on the *IL12B* rs6887695 SNP and the absence of the AA genotype on the *IL12B* rs3212227 or the GG genotype on the *IL6* rs1800795 SNP significantly increased the probability of therapeutic success in *HLA-Cw6*-positive patients [77]. Rs10484554 in the *HLA-Cw* gene did not show an association with a good response to ustekinumab in a Greek population [55]. Patients with heterozygous genotype (CT) in the *IL12B* rs3213094 showed better PASI improvement to ustekinumab than the reference genotype (CC) (∆PASI at 3 months, *p* = 0.017), but the result was not replicated with regard to PASI75 [63]. The genetic polymorphism of *TIRAP* rs8177374 and *TLR5* rs5744174 were associated with a better response in the Danish population (PASI75 at 3 months, *p* = 0.0051 and *p* = 0.0012, respectively) [58].

##### Poor Response of Efficacy

In a Spanish study that enrolled 69 patients treating psoriasis with ustekinumab, poor responders at 4 months were associated with CG/CC genotype in *CHUK* rs11591741 (*p* = 0.029), CT/CC genotype in *C9orf72* rs774359 (*p* = 0.016), AG/GG in *C17orf51* rs1975974 (*p* = 0.012), CT genotype in SLC22A4 rs1050152 (*p* = 0.037), GT/TT genotype in STAT4 rs7574865 (*p* = 0.015) and CT/CC genotype in *ZNF816A* rs9304742 (*p* = 0.012) [79]. Among 376 Danish patients, genetic variants of *IL1B* rs1143623 and rs1143627 related to increased IL-1β levels may be unfavorable outcomes (PASI75 at 3 months, *p* = 0.0019 and 0.0016, respectively), similar results with anti-TNF agents [58]. An association between the TC genotype of *IL-17F* rs763780 and no response to ustekinumab was found in 70 Spanish (PASI75 at 3 and 6 months, *p* = 0.022 and *p* = 0.016, respectively) [80]. Patients with homozygous (GG) for the rs610604 SNP in *TNFAIP3* showed a worse PASI improvement to ustekinumab (*p* = 0.031) than the TT genotype [63]. Carriers of allele G in *TNFRSF1B* rs1061622 under anti-TNF or anti-IL-12/IL-23 treatment tended to be non-responders in 90 patients from Spain (PASI < 50 at 6 months, *p* = 0.05) [59].

### 3.6. IL-17 Antagonist

Secukinumab and ixekizumab are human monoclonal antibodies that bind to the protein interleukin IL-17A, while brodalumab is a human monoclonal antibody of IL17R, which means a pan inhibitor of IL-17A, IL-17F, and IL-25. The three IL-17 antagonists are currently used in the treatment of moderate-to-severe psoriasis (Table 6).

#### 3.6.1. Secukinumab (SCK) and Ixekizumab (IXE) and Brodalumab (BDL)

##### HLA-Cw6

The responses to SCK were comparable up to 18 months between HLA-Cw*06-positive and -negative patients, as it is highly effective regardless of the HLA-Cw6 status in Italy and Switzerland [81,82,83].

##### IL-17

No associations were found between the five genetic variants of *IL-17* (rs2275913, rs8193037, rs3819025, rs7747909, and rs3748067) and ΔPASI, PASI75, or PASI90 after 12 and 24 weeks of anti-IL-17A agents, including SCK and IXE in European [84]. The lack of pharmacogenetic data for BDL was noted during the review.

### 3.7. PDE4 Antagonist

Apremilast, a selective phosphodiesterase 4 (PDE4) inhibitor, is used to treat plaque psoriasis. A Russian study identified 78 pre-selected single-nucleotide polymorphisms, increased minor allele of IL1β (rs1143633), IL4 (IL13) (rs20541), IL23R (rs2201841), and TNFα (rs1800629) genes that are associated with the better outcome in 34 patients (PASI75 at 6.5 months, *p* = 0.05, *p* = 0.04, *p* = 0.03, *p* = 0.03, respectively) [85] (Table 7).

### 3.8. Topical Agents

Globally used topical therapies for psoriasis include retinoids, vitamin D analogs, corticosteroids, and coal tar. Lack of evidence emphasizes the association between treatment response and pharmacogenetics of corticosteroids, retinoids, and coal tar. The link between *VDR* genes, encoding the nuclear hormone receptor for vitamin D3, and the response to calcipotriol has been discussed but remained controversial in different populations [86,87,88,89,90,91]. Lindioil is another topical medicine refined from Chinese herbs and is effective in treating plaque psoriasis [92]. It has been reported that HLA-Cw*06:02 positivity showed a better response (PASI75 at 3 months, *p* = 0.033) while HLA-Cw*01:02 positivity showed a poor response in 72 patients (PASI 75 at 2.5 months, *p* = 0.019) [93].

## 4. Discussion

Psoriasis has been proven to be genetically affected over half a century [94,95,96]. With the breakthrough of the technique of genetic analysis, more and more psoriasis susceptibility genes have been widely detected and analyzed as predictive markers of treatment response when unexplained and unsatisfied treatment responses and side effects have been recorded [97,98,99,100]. In addition, several reviews have highlighted the findings of pharmacogenomics in psoriasis in the last ten years [97,98,101,102,103]. In the review, regarding efficacy, carriers of HLA-Cw*06 positivity implied a more favorable response in the treatment of methotrexate and ustekinumab. *HLA-Cw6* status was not indicative of treatment response to adalimumab, etanercept, and secukinumab. Polymorphism of ABCB1 rs1045642 may indicate poor responses in Greek and Russian. However, there are some limitations in the current review. First, the relevant data of anti-IL17 agents were lacking, which reflects that it is relatively novel to the market and shows outstanding responses irrespective of genotype. Further genetic analysis of acitretin, cyclosporin, and apremilast is worth exploring. Secondly, the majority of the included pharmacogenomic studies of psoriasis were from Europe and America. This implies the limited application to Asians and Africans. It may reflect that Europe and America have more clinical trial studies or drug options, resulting in interest in studying treatment responses for psoriasis than in other areas [101]. In addition, the accessibility of gene-analysis resources may affect the development of pharmacogenomic studies. Thirdly, the protocol to identify the related gene varies between studies. A generalized and standardized method would facilitate the utilization and replication of the pharmacogenomic studies. Fourthly, pharmGKB is a comprehensive resource that curates knowledge about the impact of genetic variation on drug responses for clinicians [102]. The level of evidence of the pharmacogenetic results in this database mostly remains low (level three) due to conflicted results, small cases, or a single study. Whereas biomarkers must show a relatively strong effect in order to be of use in clinical decision-making, replicated large cohort studies of each medical therapy are required in different ethnic groups. The use of the global polygenic risk score allowed for the prediction of onset psoriasis in Chinese and Russians [85,101]. The establishment of the polygenic score for psoriasis treatment response may be developed in the future. In addition, tofacitinib, a kind of Janus kinase (JAK) inhibitor, was approved by FDA for psoriatic arthritis in 2017.

Although no indication of psoriasis alone is approved, pharmacogenetic research of JAK inhibitor is expected considering its potential cardiovascular and cancer risk in patients with rheumatoid arthritis [104].

## 5. Conclusions

This review article updates the current pharmacogenomic studies of treatment outcomes for psoriasis. A standardized protocol could be established for utilization and comparison worldwide. Currently, high-throughput whole exome sequencing (WES) or whole genome sequencing (WGS) can rapidly obtain comprehensive genetic information for individuals [105,106,107]. Genetic basic research promotes the progress of personalized medicine. Its development contributes to the precision of the effective treatment individually, providing alternatives when treatment fails, preventing adverse effects, and reducing the economic burden of treating psoriasis.

## Figures and Tables

**Table 1:** Genetic polymorphisms associated with response of methotrexate in patients with psoriasis.

Gene	SNP	Variant of Allele or Genotype (Reference Group)	Response	PharmGKB LOE	N	Population	Outcome Measures	Time Point (Month)	*p* Value	PMID
Efficacy										
*ABCC1*	rs35592	allele C (allele T)	↓	3	374	UK	PASI75, PASI < 50	3	0.008	18256692
	rs28364006	allele G (allele A)	↓	3	374	UK	PASI75, PASI < 50	3	0.02	18256692
	rs2238476	allele A (allele G)	↓	3	374	UK	PASI75, PASI < 50	3	0.02	18256692
*ABCC2*	rs717620	TT/CT (CC)	↑	NA	137	Slovenia	PASI75, PASI < 75	6	0.039	33714108
*ABCG2*	rs17731538	allele A (allele G)	↓	3	374	UK	PASI75, PASI < 50	3	0.007	18256692
	rs13120400	allele C (allele T)	↑	3	374	UK	PASI75, PASI < 50	3	0.03	18256692
*DNMT3b*	rs242913	allele T (allele C)	↓	NA	137	Slovenia	PASI75, PASI < 75	6	0.005	33714108
*FOXP3*	rs3761548	allele G (allele T)	↓	3	189	India	PASI75, PASI < 50	3	0.003	28444425
*GNMT*	rs10948059	allele T (allele C)	↓	NA	137	Slovenia	PASI75, PASI < 75	6	0.0004	33714108
*HLA-C*	Cw:06	POS (NEG)	↑	3	189	India	PASI75, PASI < 50	3	0.004	28444425
		POS (NEG)	↑	NA	70	UK	Treatment duration beyond 12 months or not	12	0.05	28512993
*MTHFR*	rs1801131	CT (TT/CC)	↓	NA	309	Chinese	PASI75, PASI < 75	3	0.014	35479943
	rs1801133	TT (CT/CC)	↑	NA	309	Chinese	PASI90, PASI < 90	3	0.006	35479943
*SLCO1B1*	-	Low haplotype activity (High)	↑	NA	137	Slovenia	PASI75, PASI < 75	6	0.027	33714108
*TNIP1*	rs10036748	TT (CC)	↑	NA	221	Chinese	PASI75, PASI < 75	3	0.043	31020648
Toxicity										
*ABCC1*	rs246240	allele G (allele A)	↓	3	374	UK	susceptible to toxicity	-	0.0006	18256692
	rs2238476	allele A (allele G)	↓	3	374	UK	susceptible to toxicity	-	0.01	18256692
	rs1967120	allele A (allele G)	↓	NA	374	UK	susceptible to toxicity	-	0.01	18256692
	rs11075291	allele A (allele G)	↓	NA	374	UK	susceptible to toxicity	-	0.008	18256692
	rs3784862	Allele G (allele A)	↓	NA	374	UK	susceptible to toxicity	-	0.002	18256692
	rs3784864	Allele A (allele G)	↓	NA	374	UK	susceptible to toxicity	-	0.03	18256692
*ATIC*	rs2372536	No specific genotype	↑	NA	188	UK	discontinuation due to AE	-	0.038	17410198
		Allele G (allele C)	↓	NA	374	UK	susceptible to toxicity	-	0.01	19016697
	rs4672768	c.1660-135G>A Homozygotes for the major allele	↑	NA	374	UK	susceptible to toxicity	-	0.02	19016697
*ADORA2A*	rs5760410	G > A Homozygotes for the major	↑	NA	374	UK	susceptible to toxicity	-	0.03	18256692
*BHMT*	rs3733890	AA/GA (GG)	↑	NA	137	Slovenia	Hepatotoxicity	-	0.022	33714108
*MTHFR*	rs1801131	CT (TT/CC)	↓	NA	309	Chinese	Hepatotoxicity	-	0.04	35479943
*SLC19A1*	rs1051266	Allele A (Allele G)	↑	NA	188	UK	Occurrence of AE	-	0.025	17410198
		Allele A (Allele G)	↑	NA	374	UK	Associated with toxicity	-	0.03	18256692
*TYMS*	rs11280056	6bp del allele (wild allele)	↑	4	188	UK	Occurrence of AE	-	0.025	17410198
	rs34743033	3R allele (wild allele)	↑	NA	188	UK	Toxicity when not receiving folic acid	-	0.0025	17410198

Abbreviations: NA, not applicable; N, number; PASI, psoriasis area severity index; PharmGKB LOE, pharmacogenomics knowledge base level of evidence; PMID, PubMed unique identifier; SNP, single nucleotide polymorphism; UK, United Kingdom; ↑, increased efficacy or toxicity; ↓, decreased efficacy or toxicity.

**Table 2:** Genetic polymorphisms associated with response of acitretin in patients with psoriasis.

Gene	SNP	Variant of Allele or Genotype (Reference Group)	Response	PharmGKB LOE	N	Population	Outcome Measures	Time Point (Month)	*p* Value	PMID
Efficacy										
*ANKLE1*	rs11086065	AG/GG (AA)	↓	NA	166	Chinese	PASI75	3	0.003	28146080
*ARHGEF*	rs3821414	CT/CC (TT)	↑	NA	166	Chinese	PASI75	3	0.01	
*CRB2*	rs1105223	TT/CT (CC)	↑	NA	166	Chinese	PASI75	3	0.048	
*HLA-DQ*	DQA1*02:01	POS (NEG)	↑	NA	100	Chinese	PASI75	2	0.001	36224009
*HLA-DQ*	DQB*02:02	POS (NEG)	↑	NA	100	Chinese	PASI75	2	0.005	
*HLA-G*		14 bp del allele (−)	↑	NA	21	Italy	PASI75	4	0.008	24909182
*IL-12B*	*rs3212227*	TG	↑	NA	43	Chinese	PASI50	NA	0.035	35814239
*IL-23R*	*rs112009032*	AA	↑	NA	43	Chinese	PASI75	NA	0.02	
*SFRP4*	rs1802073	GG/GT (TT)	↑	NA	166	Chinese	PASI75	3	0.007	28146080
*VEGF*	rs833061	TT (−)	↓	NA	106	UK	PASI75, PASI < 50	3	0.04	16385345
		TC (−)	↑	NA	106	UK	PASI75, PASI < 50	3	0.01	

Abbreviations: NA, not applicable; N, number; PASI, psoriasis area severity index; PharmGKB LOE, pharmacogenomics knowledge base level of evidence; PMID, PubMed unique identifier; SNP, single nucleotide polymorphism; UK, the United Kingdom;↑, increased efficacy or toxicity; ↓, decreased efficacy or toxicity.

**Table 3:** Genetic polymorphisms associated with response of cyclosporin in patients with psoriasis.

Gene	SNP	Variant of Allele or Genotype (Reference Group)	Response	PharmGKB LOE	N	Population	Outcome Measures	Time Point (Month)	*p* Value	PMID
Efficacy										
*ABCB1*	rs1045642	Allele T (allele C)	↓	NA	84	Greece	PASI75, PASI < 50	3	0.007	24889923
*ABCB1*	rs1045642	TT/CT (CC)	↓	NA	168	Russian	PASI75, PASI < 50	3	<0.001	36432633
*ABCB1*	rs1128503	TT/CT (CC)	↓	NA	168	Russian	PASI75, PASI < 50	3	0.027	
*ABCB1*	rs2032582	TT/GT (GG)	↓	NA	168	Russian	PASI75, PASI < 50	3	0.048	
*ABCB1*	Block1	TGC haplotype	↓	NA	168	Russian	PASI75, PASI < 50	3	<0.001	
*CALM1*	rs12885713	Allele T (allele C)	↑	NA	200	Greece	PASI75, PASI < 50	3	0.011	36229649
*MALT1*	rs2874116	Allele G (allele A)	↑	NA	200	Greece	PASI75, PASI < 50	3	<0.001	

Abbreviations: NA, not applicable; N, number; PASI, psoriasis area severity index; PharmGKB LOE, pharmacogenomics knowledge base level of evidence; PMID, PubMed unique identifier; SNP, single nucleotide polymorphism; ↑, increased efficacy or toxicity; ↓, decreased efficacy or toxicity.

**Table 4:** Genetic polymorphisms associated with response of TNF antagonist in patients with psoriasis.

Gene	SNP	Variant of Allele or Genotype (Reference Group)	Drug	Response	PharmGKB LOE	N	Population	Outcome Measures	Time Point (Month)	*p* Value	PMID
Efficacy											
*CD84*	rs6427528	GA (GG)	ETN	↑	3	161	Netherlands	∆PASI	3	0.025	27564082
*CDKAL1*	rs6908425	CT/TT (CC)	Anti-TNF	↑	3	133	Spain	PASI75	6	0.013	27670765
*CTNNA2*	rs11126740	AG/GG (AA)	Anti-TNF	↑	3	144	Spain	PASI 75	3	0.003	27670765
*CPM*	CNV	3.5 × 10^5^ bp	ADA	↑	NA	70	Spain	PASI90, PASI < 75	3 and 6	<0.05	338466759
*FCGR2A*	rs1801274	AA/AG (GG)	ADA, ETN, IFX	↑	4	70	Spain	BSA	2	0.03	24048425
				-	NA	70	Spain	∆PASI, PASI 75	3	0.18	24048425
				-	NA	100	Greece	PASI75	6	0.749	27044681
*FCGR3A*	rs396991	GG/GT (TT)	ADA, ETN, IFX	↑	3	100	Greece	PASI75	6	0.005	27044681
			ETN	↑		55				0.01	
			ADA, IFX	-		45				0.331	
		AA (AC/CC)	ADA, ETN, IFX	↓	3	70	Spain	BSA	2	0.02	24048425
				-	NA		Spain	PASI 75	3	0.13	
*HLA-A*	rs9260313	TT (CT/CC)	ADA	↑	NA	39	UK	PASI75, PASI < 50	6	0.05	27043841
*HLA-C*	Cw6	POS (NEG)	ADA	↑	NA	169	Spain	PASI75	6	0.018	31267486
*HLA-C*	rs12191877	CT/TT (CC)	Anti-TNF	↑	3	144	Spain	PASI 75	3	0.05	27670765
*HLA-C*	rs10484554	CT/CC (TT)	Anti-TNF	↑	NA	199	UK	PASI75, PASI < 50	6	0.032	27043841
*IL12B*	rs2546890	AG/GG (AA)	Anti-TNF	↓	3	144	Spain	PASI 75	3	0.017	27670765
*IL17RA*	rs4819554	Allele A (allele G)	Anti-TNF	↑	3	238	Spain	PASI 75	3	0.01	27670766
					NA			PASI 75	6	0.04	
		AA (AG/GG)	Anti-TNF	↑	NA	238	Spain	PASI 75	3	0.03	27670766
*IL1B*	rs1143623	CG/GG (CC)	Anti-TNF	↓	3	376	Denmark	PASI75, PASI < 50	3	0.0041	28696418
	rs1143627	AG/GG (AA)	Anti-TNF	↓	3	376	Denmark	PASI75, PASI < 50	3	0.0016	28696418
*IL-17F*	rs763780	TC (TT)	ADA	↓	NA	67	Spain	PASI75	6	0.0044	26415694
		TC (TT)	IFX	↑	NA	37	Spain	PASI75	3	0.023	26415694
		TC (TT)	IFX	↑	NA	37	Spain	PASI75	6	0.02	26415694
*IL23R*	rs11209026	GG	Anti-TNF	↑	NA	109	Spain	PASI90	6	0.006	23662788
*LY96*	rs11465996	Allele G (allele C)	Anti-TNF	↓	3	376	Denmark	PASI75, PASI < 50	3	0.0044	28696418
*MAP3K1*	rs96844	AG/GG (AA)	Anti-TNF	↑	3	144	Spain	PASI75	3 and 6	0.004	27670765
*NFKBIZ*	rs3217713	Deletion	ADA	↑	NA	169	Spain	PASI75	6	0.015	31267486
*PGLYRP4*	rs2916205	CC/CT (TT)	Anti-TNF	↓	3	144	Spain	PASI75	3	0.05	27670765
*TLR2*	rs4696480	AT/TT (AA)	Anti-TNF	↓	3	376	Denmark	PASI75, PASI < 50	3	0.0032	28696418
*TLR2*	rs11938228	AA/AC (CC)	Anti-TNF	↓	3	376	Denmark	PASI75, PASI < 50	3	0.0019	28696418
*TLR9*	rs352139	CT/TT (CC)	Anti-TNF	↑	3	376	Denmark	PASI75, PASI < 50	3	0.0017	28696418
*TNF*	rs361525	GG	Anti-TNF	↑	NA	109	Spain	PASI75	6	0.049	23662788
*TNF*	rs1799724	CT/TT	Anti-TNF	↑	NA	109	Spain	PASI75	6	0.006	23662788
		CT/TT (TT)	ETN	↑	NA	80	Greece	PASI75, PASI < 50	6	0.002	22111980
*TNF*	rs1799964	TT	Anti-TNF	↑	NA	109	Spain	PASI75	6	0.038	23662788
*TNFAIP3*	rs610604	AA/CA (CC)	ETN	↑	NA	35	UK	PASI75, PASI < 50	6	0.007	27043841
*TNFRSF1B*	rs1061622	TT/TG (GG)	ETN	↑	NA	80	Greece	PASI75, PASI < 50	6	0.001	22111980
*TNFRSF1B*	rs1061622	Allele G (allele T)	Anti-TNF	↓	NA	53	Spain	PASI75	6	0.03	25537528
*TRAF3IP2*	rs13190932	GG (GA + AA)	IFX	↑	NA	124	UK	PASI75, PASI < 50	6	0.041	27043841
*ZNF816A*	rs9304742	CC (CT + TT)	Anti-TNF	↓	3	144	Spain	PASI75	3	0.02	27670765
*Toxicity*											
*ARNT2*, *LOC101929586*, *MIR5572*	CNV	1 × 10^5^ bp	ADA, IFX, ETN	↑	NA	70	Spain	PP	3 and 6	0.006	33846759
*CTLA4*	rs3087243	AG/GG (AA)	Anti-TNF	↓	3	161	Spain	PP	9	0.005	26194362
*FBXL19*	rs10782001	GG (AA/AG)	Anti-TNF	↑	3	161	Spain	PP	9	0.028	26194362
*IL23R*	rs11209026	AG (GG)	Anti-TNF	↑	3	161	Spain	PP	9	0.012	26194362
*SLC12A8*	rs651630	TT (AA/AT)	Anti-TNF	↓	3	161	Spain	PP	9	0.011	26194362
*TAP1*	rs1135216 (former rs1800453)	AG (AA/GG)	Anti-TNF	↓	-	161	Spain	PP	9	0.018	26194362

Abbreviations: ADA, Adalimumab; ETN, Etanercept; IFX, Infliximab; NA, not applicable; N, number; PASI, psoriasis area severity index; PharmGKB LOE, pharmacogenomics knowledge base level of evidence; PMID, PubMed unique identifier; PP, paradoxical psoriasiform reaction.; SNP, single nucleotide polymorphism; TNF, Tumor necrosis factor; UK, the United Kingdom; ↑, increased efficacy or toxicity; ↓, decreased efficacy or toxicity.

**Table 5:** Genetic polymorphisms associated with response of IL-12/IL-23 antagonist in patients with psoriasis.

Gene	SNP	Variant of Allele or Genotype (Reference Group)	Drug	Response	PharmGKB LOE	N	Population	Outcome Measures	Time Point (Month)	*p* Value	PMID
Efficacy											
*ADAM33*	rs2787094	CC	UTK	↑	NA	69	Spanish	PASI75	4	0.015	27977334
*CHUK*	rs11591741	CG/CC	UTK	↓	NA	69	Spanish	PASI75	4	0.029	
*C9orf72*	rs774359	CT/CC	UTK	↓	NA	69	Spanish	PASI75	4	0.016	
*C17orf51*	rs1975974	AG/GG	UTK	↓	NA	69	Spanish	PASI75	4	0.012	
*ERAP1*	rs26653	GC/GG (CC)	UTK	↑	NA	22	UK	PASI75, PASI < 50	6	0.016	27043841
	rs151823	CC (CA)	UTK	↑	NA	22	UK	PASI75, PASI < 50	6	0.026	
*HLA-C*	Cw*06:02	POS (NEG)	UTK	↑	NA	332	USA	PASI75	3	<0.05	27476722
			UTK	↑	NA	937	Netherlands	PASI75	6	<0.001	30994858
			UTK	↑	NA	255	Italy	PASI50	1	<0.0001	28207934
	Cw*06	POS (NEG)	UTK	↑	3	51	Italy	PASI75	3	<0.008	23521149
			UTK	↑	NA	66	Taiwan	PASI75	7	0.019	24734995
			UTK	↑	NA	134	Italy	PASI75	3	0.001	26775778
			UTK	↑	NA	64	Italy	PASI75	7	0.028	26678060
*HLA-C*, *IL12B*	Cw*06, rs6887695	POS, GG (NEG, CG/CC)	UTK	↑	NA	64	Italy	PASI75	7	0.033	
	Cw*06, rs3212227	POS, CA/CC (NEG, AA)	UTK	↑	NA	64	Italy	PASI75	7	0.034	
*HLA-C*, *IL6*	Cw*06, rs1800795	POS, CG/CC (NEG, GG)	UTK	↑	NA	64	Italy	PASI75	7	0.026	
*HTR2A*	rs6311	CT/TT	UTK	↑	NA	69	Spanish	PASI75	4	0.037	27977334
*IL1B*	rs1143623	CG/GG (CC)	UTK	↓	3	376	Denmark	PASI75, PASI < 50	3	0.0019	28696418
	rs1143627	AG/GG (AA)	UTK	↓	3	376	Denmark	PASI75, PASI < 50	3	0.0016	
*IL12B*	rs3213094	CT (CC)	UTK	↑	3	66	Netherlands	∆PASI	3	0.017	27564082
*IL-13*	rs848	GT/TT	UTK	↑	NA	69	Spanish	PASI75	4	0.037	27977334
*IL-17F*	rs763780	TC (TT)	UTK	↓	NA	70	Spain	PASI75	3	0.022	26415694
									6	0.016	
*NFKBIA*	rs2145623	CC	UTK	↑	NA	69	Spanish	PASI75	4	0.024	27977334
*SLC22A4*	rs1050152	CT	UTK	↓	NA	69	Spanish	PASI75	4	0.037	27977334
*STAT4*	rs7574865	GT/TT	UTK	↓	NA	69	Spanish	PASI75	4	0.015	27977334
*TIRAP*	rs8177374	CT/TT (CC)	UTK	↑	3	230	Denmark	PASI75, PASI < 50	3	0.0051	28696418
*TLR5*	rs5744174	AG/GG (AA)	UTK	↑	3	230	Denmark	PASI75, PASI < 50	3	0.0012	28696418
*TNFAIP3*	rs610604	GG (TT)	UTK	↓	4	66	Netherlands	∆PASI	3	0.031	27564082
		GG (TT)	UTK	-	4	51	Italy	PASI75	3	0.75	23521149
*TNFR1*	rs191190	CT/CC	UTK	↑	NA	69	Spanish	PASI75	4	0.037	27977334
*TNFRSF1B*	rs1061622	Allele G (allele T)	UTK	↓	NA	8	Spain	PASI75, PASI < 50	6	0.05	25537528
*ZNF816A*	rs9304742	CT/CC	UTK	↓	NA	69	Spanish	PASI75	4	0.012	27977334

Abbreviations: NA, not applicable; N, number PASI, psoriasis area severity index; PharmGKB LOE, pharmacogenomics knowledge base level of evidence; PMID, PubMed unique identifier; SNP, single nucleotide polymorphism; UK, the United Kingdom; UTK, Ustekinumab;↑, increased efficacy or toxicity; ↓, decreased efficacy or toxicity.

**Table 6:** Genetic polymorphisms associated with response of IL-17 antagonist in patients with psoriasis.

Gene	SNP	Variant of Allele or Genotype (Reference Group)	Drug	Response	PharmGKB LOE	N	Population	Outcome Measures	Time Point (Month)	*p* Value	PMID
Efficacy											
*HLA-C*	HLA-Cw6	Cw*06-POS (Cw*06-NEG)	SCK	-	NA	434	Italy	PASI90	4	0.293	29704432
*HLA-C*	HLA-Cw6	Cw*06-POS (Cw*06-NEG)	SCK	-	NA	434	Italy	PASI90	18	>0.05	31001812
*HLA-C*	HLA-Cw6	Cw*06-POS (Cw*06-NEG)	SCK	-	NA	18	Switzerland	∆PASI	3	>0.05	29356172
*IL-17*	rs2275913 rs8193037 rs3819025 rs7747909 rs3748067	GA/AA (GG) GA/AA (GG) GA/AA (GG) GA/AA (GG) CT/TT (CC)	SCK, IXE	-	NA	134	Italy	∆PASI	3 and 6	>0.05	31287604

Abbreviations: IXE, Ixekizumab; NA, not applicable; N, number; PASI, psoriasis area severity index; PharmGKB LOE, pharmacogenomics knowledge base level of evidence; PMID, PubMed unique identifier; SCK, Secukinumab; SNP, single nucleotide polymorphism.

**Table 7:** Genetic polymorphisms potentially associated with response of apremilast in patients with psoriasis.

Gene	SNP	Variant of Allele or Genotype (Reference Group)	Response	PharmGKB LOE	N	Population	Outcome Measures	Time Point (Month)	*p* Value	PMID
Efficacy										
*IL-1B*	rs1143633	T (C)	↑	NA	34	Russian	PASI75	6.5	0.05	33383665
*IL-4*	rs20541	A (G)	↑	NA	34	Russian	PASI75	6.5	0.04	33383665
*IL-23R*	rs2201841	G/T (A)	↑	NA	34	Russian	PASI75	6.5	0.03	33383665
*TNF-a*	rs1800629	A (G)	↑	NA	34	Russian	PASI75	6.5	0.03	33383665

Abbreviations: NA, not applicable; N, number; PASI, psoriasis area severity index; PharmGKB LOE, pharmacogenomics knowledge base level of evidence; PMID, PubMed unique identifier; SNP, single nucleotide polymorphism; ↑, increased efficacy or toxicity.

## Data Availability

Not applicable.

## Supplementary Materials

The following supporting information can be downloaded at: https://www.mdpi.com/article/10.3390/ijms24087329/s1.
