# Supplementary material for: Pharmacogenomics on the Treatment Response in Patients with Psoriasis: An Updated Review
Source: Int J Mol Sci. 2023 Apr 15;24(8):7329. doi: 10.3390/ijms24087329 (PMC10138383; doi:10.3390/ijms24087329)
Supplement: Supplementary file 1 [file ijms-24-07329-s001.zip › ijms-2167000-supplementary.pdf]

Supplementary Table S1. Brief summary of drugs mentioned in the article

Classification	Drug	Route of administration
DMARD	Methotrexate	Oral
Retinoid	Acitretin	Oral
Calcineurin inhibitor	Cyclosporin	Oral
TNF antagonist	Adalimumab	Subcutaneous
	Etanercept	Subcutaneous
	Infliximab	Intravenous
IL-12/IL-23 antagonist	Ustekinumab	Subcutaneous
IL-17 antagonist	Brodalumab	Subcutaneous
	Ixekizumab	Subcutaneous
	Secukinumab	Subcutaneous
PDE4 antagonist	Apremilast	Oral
Vitamin D analogue	Calcipotriol	Topical
Indigo Naturalis	Lindioil	Topical

DMARD, disease-modifying anti-rheumatic drug; IL-12, interleukin-12; IL-17, interleukin-17; IL-23, interleukin-23; PDE4, phosphodiesterase-4; TNF, tumor necrosis factor
